# Repetitive Transcranial Magnetic Stimulation: a Novel Approach for Treating Oropharyngeal Dysphagia

**DOI:** 10.1007/s11894-015-0483-8

**Published:** 2016-02-20

**Authors:** Emilia Michou, Alicja Raginis-Zborowska, Masahiro Watanabe, Taha Lodhi, Shaheen Hamdy

**Affiliations:** Centre for Gastrointestinal Sciences, Institute of Inflammation and Repair, Faculty of Medical and Human Sciences, University of Manchester, Clinical Sciences Building, Salford Royal Hospital (part of the Manchester Academic Health Sciences Centre (MAHSC)), Eccles Old Road, Salford, M6 8HD UK

**Keywords:** Swallowing disorders, Rehabilitation, Neurostimulation, Brain, Neurophysiology

## Abstract

In recent years, repetitive transcranial magnetic stimulation, a technique used to produce human central neurostimulation, has attracted increased interest and been applied experimentally in the treatment of dysphagia. This review presents a synopsis of the current research for the application of repetitive transcranial magnetic stimulation (rTMS) on dysphagia. Here, we review the mechanisms underlying the effects of rTMS and the results from studies on both healthy volunteers and dysphagic patients. The clinical studies on dysphagia have primarily focussed on dysphagia post-stroke. We discuss why it is difficult to draw conclusions for the efficacy of this neurostimulation technique, given the major differences between studies. The intention here is to stimulate potential research questions not yet investigated for the application of rTMS on dysphagic patients prior to their translation into clinical practice for dysphagia rehabilitation.

## Introduction

Deglutition is one of the most important bodily functions, allowing the intake of required nutrients and hydration. The ability to swallow safely is of importance, since the consequences of unsafe swallowing (dysphagia) can directly threaten an individual’s well-being.

Currently, the clinical guidelines for the management of dysphagic patients constitute mainly of compensatory strategies or postural changes to try and prevent complications [1]. Delivered by speech and language therapists, dysphagia rehabilitation approaches include a variety of head and neck exercises (chin tuck, head turn or Mendelsohn manoeuvre) but with little limited evidence to support their efficacy [1, 2].

The therapeutic procedures for oropharyngeal dysphagia have changed dramatically mainly due to advances in medical experimental imaging and neurostimulation along with our knowledge on the neurophysiological properties of deglutition.

Here, we will briefly discuss the neurophysiological underpinnings of deglutition before examining recent advances in a new therapeutic neurostimulation technique for dysphagia, namely repetitive transcranial magnetic stimulation (rTMS), which has attracted increased interest over the past decade.

## Neurophysiology of Deglutition

Deglutition is the output of a very precise multidimensional interplay between different brain areas, translated into a well-tuned coordinated muscle activity in the periphery.

Historically, the central neural control of swallowing was believed to be almost entirely dependent on brainstem reflexive mechanisms [3]. However, in recent years, the role of the cerebral cortex in swallowing has received increased recognition and has been the subject of much research [4, 5].

Much of our understanding of the neural control of swallowing has come from invasive neurophysiological observations in animals [6], replicated by many other groups in differing animal species [3, 7–14]. Artificially stimulating cortical swallowing areas using invasive electrical microstimulation of either cortical hemisphere in anaesthetised animals is capable of inducing full swallow responses, which provided evidence that swallowing musculature is bilaterally controlled over the cortical level. In humans, neural cartographer and neurosurgeon Wilder Penfield and colleagues, using the same techniques of invasive electrical microstimulation in anaesthetised patients undergoing neurosurgery, demonstrated that stimulation to certain parts of the cerebral cortex could also induce swallowing [15].

One of the first non-invasive studies of swallowing conducted in dogs showed that with the use of transcranial magnetic stimulation (TMS), activation of the cerebral cortex through the scalp surface could elicit full swallowing responses [16].

Nowadays, a number of TMS techniques are used for routine diagnostic application in neurophysiological settings [17, 18]. TMS is a safe and non-invasive technique which uses a high-current pulse generator discharging currents of several thousand amperes that flow through a coil of wire. The result is the generation of a brief magnetic pulse with field strengths up to several Tesla. When the coil is placed over the subject’s head, the magnetic field undergoes little attenuation by extracerebral tissues (scalp, cranial bone, meninges and cerebrospinal fluid layer) and induces an electrical field sufficient to depolarise superficial axons and to activate cortical neural networks. Several physical and biological parameters play a role in the outcome of the stimulation, such as the type and orientation of coil; the distance between the coil and the brain; the magnetic pulse waveform; and the intensity, frequency and pattern of stimulation [19]. Perpendicular currents of sufficient strength are generated to depolarise neuronal elements and evoke electromyographic responses on the targeted musculature, called motor evoked potentials (MEPs).

With TMS, the midline structures involved in swallowing, mylohyoid, pharyngeal and oesophageal musculature were mapped in healthy volunteers by Hamdy and colleagues [20]. In health, human swallowing musculature in the cerebral cortex was shown to be discretely and somatotopically represented bilaterally (motor and premotor cortices) with a marked display of interhemispheric asymmetry, independent of handedness, thereby inferring the presence of ‘dominant’ and ‘non-dominant’ hemispheres for the task of swallowing.

In the recent years, neuroimaging and neurostimulation studies have provided insights into the activation patterns of the swallowing sequence and muscle activities (for reviews [4, 21]) and verified the earlier results. An activation likelihood estimation meta-analysis of imaging studies on swallowing [22] showed that the most consistent areas that are activated in these neuroimaging studies include the primary sensorimotor cortex (M1/S1), sensorimotor integration areas, the insula and frontal operculum, the anterior cingulate cortex and supplementary motor areas (SMAs). Recently, Mihai et al. [23] using dynamic causal modelling examined the potential effective connectivity of areas such as SMA, M1/S1 and insula during swallowing and showed that there is high probability of bidirectional connections of the areas such as the SMA and M1/S1 during swallowing. In addition, the cerebellum, important in planning and executing complex motor tasks, has been strongly implicated in the neurophysiological control of swallowing, both through animal studies [24] and human functional brain imaging [25–32] and TMS studies as described below.

Recently, TMS has been used to study the role of cerebellum in swallowing. Jayasekeran et al. [33] systematically probed this relationship using single-pulse TMS and discovered that distinctive cerebellar-evoked pharyngeal motor evoked potentials with similar response latencies to cortically evoked (cortical) PMEPs could be evoked from cerebellar sites (both the cerebellar midline and hemispheres). Interestingly, when paired pulses of cerebellar–cortical conditioning were delivered at short interstimulus intervals (ISIs) (50, 100 and 200 ms), this strongly excited pharyngeal corticobulbar projections [33].

## Dysphagia and Plasticity

Following a focal brain lesion such as stroke, patients may experience swallowing disorders (dysphagia), a devastating complication resulting in increased risk of aspiration pneumonia [34–36]. Evidence exists for the effective recovery of swallowing function after unilateral stroke, which is associated with increase in cortical excitability and cortical area map size of the unaffected hemisphere [37–39]. In a seminal study of swallowing in stroke using TMS, both dysphagic and non-dysphagic patients had the cortical topography of their pharyngeal musculature serially mapped over several months [20]. A follow-up study [37] showed that the cortical map representation of the pharyngeal musculature in the undamaged hemisphere markedly increased in size in dysphagic patients who recovered swallowing, whilst there was no change in patients who had persistent dysphagia or in patients who were non-dysphagic throughout. Furthermore, changes seen in the damaged hemisphere in any of the groups of patients were not significant. These observations implied that over a period of weeks or months, the recovery of swallowing after stroke may be reliant on compensatory strategies of cortical reorganisation, through neuroplastic changes, mainly observed in the undamaged hemisphere.

Given this increase in our knowledge on swallowing neurophysiology and pathophysiology, there is now a plethora of stimulus-driven neuroplasticity protocols being trialled in order to augment and accelerate these cortical changes in dysphagic patients [21, 40].

## Repetitive TMS and Underlying Mechanisms

In the recent years, rTMS has become widely used in the form of two treatment regimens: low-frequency rTMS, which is defined by stimulation at frequencies lower than or equal to 1 Hz, and high-frequency rTMS, which is defined by stimulation at frequencies higher than or equal to 5 Hz. Low-frequency rTMS reduces neuronal excitability, whereas high-frequency rTMS increases cortical excitability [41].

A number of randomised placebo-controlled studies have generally demonstrated that rTMS efficaciously treats a variety of pathological conditions and diseases such as stroke, depression, tinnitus, obsessive–compulsive disorders, pain syndromes, migraines, refractory epilepsy, dystonia, tremors and spasticity (for reviews, see [19, 42] and [43]). In an extensive evidence-based synthesis of established and potential therapeutic applications of rTMS, Lefaucher and colleagues [19] concluded that level A recommendation has been achieved so far for the beneficial effect of high-frequency rTMS on neuropathic pain (target: M1 contralateral to pain side) and major depression but highlighted the fact that more controlled studies should take place to verify the utility while controlling for factors as time of introduction of the treatment and concurrent pharmacological interventions.

However, although numerous studies have investigated the effects of TMS and found beneficial effects, two primary issues remain unclear: first, the underlying mechanisms for the induction of changes following rTMS in such a range of diseases and, secondly, why are there long-lasting changes manifested and what are the mechanisms behind maintenance of the effects (usually the effects last more than 6 months).

Chervyakov et al. [43] reviewed the various potential mechanisms relative to the actions of TMS at neural network (mutual exCitation and inhibition of cerebral regions), synaptic and/or molecular genetic (changes in gene expression, enzyme activity and neuromediator production) levels. One of the most important mechanisms underlying the changes following rTMS is now considered the change in neurotransmitter concentrations following rTMS, such as endogenous dopamine [44, 45].

Moreover, results from research studies employing rTMS have reported some dependence of benefits from TMS and genetic polymorphisms [46–48].

Similar to all brain neurostimulation techniques, several parameters play a role for the effective application of rTMS application, including coil orientation, coil type, target selection, distance to target (from the maximum output of magnetic field to the brain area target for stimulation) and specific parameters such as intra-train interval, pulse width, frequency of the pulses, duration of the stimulation protocol and intensity used to deliver the stimulation. Worth mentioning is that the repetition of application within a protocol (treatment regimen) as well as factors such as time of the day (circadian rhythms) and brain activation state prior to treatment can play a role in the outcome [49].

## Repetitive TMS in Health-Effective Deglutition

Studies for the effects for rTMS in healthy subjects usually serve as a prelude to the application of the techniques to patients with dysphagia. Gow et al. [50] explored the effects of 100 pulses of rTMS over the pharyngeal motor cortex (80 % pharyngeal threshold) and observed an increase in cortical excitability lasting for over 1 h using a 5-Hz frequency. Comparing the effects of different number of pulses in trains of 5 Hz, Jefferson et al. [51] found that 250 pulses were as effective as longer 5-Hz rTMS trains (1000 pulse) at inducing increase in cortico-bulbar MEPs from pharyngeal M1. Conversely, Mistry et al. [52] showed that with an inhibitory, 1-Hz rTMS paradigm for 10 min (600 magnetic stimulation pulses) at the 120 % of pharyngeal threshold was possible to generate a unilateral ‘virtual lesion’, inhibition of cortico-bulbar output, in the pharyngeal motor cortex for up to 45 min and can also interfere with swallowing behaviour, as measured using reaction time swallowing tasks.

Apart from MEPs, reaction time swallowing tasks, where the subject has to perform a swallow within a specified time window as measured by intra-pharyngeal manometry, have been also used to examine the effects of 5-Hz excitatory stimulation following the inhibition induced with 1 Hz rTMS in healthy subjects [51]. The rationale behind these research studies was to attempt to interfere temporarily with neuronal function and inhibit the area of interest (with 1 Hz) on the hemispheric ‘dominant’ side, thus simulating the effects of a lesion. Thereafter, 5 Hz was delivered to both the pharyngeal M1 hotspots ipsilateral or contralateral to the lesion on different occasions (as well as no stimulation, control arm). Comparing the effects of between the two different target locations, it was shown that when the 5 Hz was applied contralateral to the virtual lesion, the inhibitory effects of the latter were reversed. This non-competitive synergy between the two pharyngeal M1s has been verified with other studies recently [53, 54] following the similar translational model of virtual lesion with outcome measures the changes in MEPs or SRTs.

Moreover, Verin et al. [55] have used videofluoroscopy to examine the effects of 1 Hz rTMS on oropharyngeal motor cortex and observed a transient change in swallowing behaviour in a way reminiscent to that seen in stroke patients with hemispheric lesions.

Recently, Vasant and colleagues [56••] examined the effects of differing frequencies of cerebellar rTMS on pharyngeal cortical and cerebellar excitability. High-frequency cerebellar rTMS (10 Hz) can robustly produce physiologically relevant effects on the excitability of frequency specific of corticobulbar projections to the pharynx. Of interest and as before, these effects were frequency specific, and with the advantage of neuronavigation, the authors were able to confirm the optimal posterior fossa sites where stimulation can be applied to modulate pharyngeal corticobulbar excitability and swallowing responses.

## Repetitive TMS in Dysphagia

In deglutition, rTMS has been studied vastly over the last few years as means to either augment or as a treat-alone avenue for dysphagia rehabilitation. The effects of rTMS have been studied mostly in healthy adults and stroke patients in studies where various outcome measures were employed. There are now two published systematic reviews and meta-analyses for brain stimulation in dysphagia, where studies with rTMS on stroke patients with dysphagia were included [57, 58]. Table 1 presents all the studies where rTMS was performed to dysphagic patients.

**Table 1:** Published studies with the oldest first

Study	Demographics		Study design	Parameters						Results/comments
	Total *n* participants	Characteristics		Stimulation	Design	Hemisphere	Location (motor cortex)	Coil size	Schedule	
[60]	26 (10 male) 57.3 ± 12 yoa	Acute hemispheric stroke	RCT (rTMS vs. sham)	3 Hz rTMS (120 % rMT)	10 blocks of 30 pulses	Affected	Oesophageal	90 mm figure 8	5 days, 10 min/day	Real rTMS increased MEP amplitude bilaterally, decrease dysphagia severity degree (self-rated)
[55]	7 (4 male) 65 ± 10 yoa	Hemispheric or sub-hemispheric	Uncontrolled case series	1 Hz rTMS (120 % rMT)	1 block	Unaffected	Mylohyoid	70 mm figure 8	20 min, once a day, 5 days	Real rTMS reduced swallowing reaction time on VFS (liquids and paste boluses), the AP scores with liquids and the residue score paste
[63]	22 (16 male) LMI group: 56 ± 15 yoa BI: 58 ± 10 yoa	LMI = 11, BI = 11	Controlled design	3 Hz rTMS (130 % rMT unaffected)	10 blocks of 30 pulses	Bilateral	Oesophageal	90 mm figure 8	5 days, 10 min/day	Both groups reduced dysphagia severity degree (self-rated). Results maintained over 2 months.
[65]	30 (17 male) 68.2 ± 1 yoa	Infarct (*n* = 15), haemorrhage (*n* = 13), TB I (*n* = 2)	RCT (2 rTMS arms vs. Control)	5 Hz rTMS (100 % rMT)	20 blocks of 50 pulses	Affected	Mylohyoid ‘hot spot’	90 mm figure 8	10 days, 20 min/day	1Hz rTMS improved functional dysphagia scale and AP scores
				1 Hz rTMS (100 % rMT)	1 block of 1200 pulses	Unaffected				
				Sham		Affected				
[61••]	18 (10 male) 71 ± 7 yoa	3 haemorrhage, 15 infarction	RCT (treatment vs. Control)	5 Hz rTMS (90 % rMT)	10 blocks of 50 pulses	Unaffected	Pharyngeal	70 mm figure 8	10 days, 10 min/day	Real rTMS reduced AP scores and residue
[66••]	18 (15 male) 66 ± 3 yoa	Hemispheric and sub-hemispheric	RCT (3 arms) T1: rTMS T2: PES T3: PAS	5 Hz rTMS (90 % rMT)	5 blocks of 50 pulses	Unaffected	Pharyngeal	70 mm figure 8	Single	No significant difference between real and sham for cortical excitability and no difference in cumulative AP scores
[62]	4 (2 male) 56–80 yoa	Bilateral stroke	Uncontrolled case series	3 Hz rTMS (130 % rMT)	Twice × 300 LH and 300 RH, total 1200 pulses/day	Bilateral	Pharyngeal	70 mm figure 8	6 day	Reduced AP score in three fourths of patients
[64]	Total: 47 rTMS arm, *n* = 14 (6 males) 59.8 ± 11 yoa	Unilateral hemispheric stroke	Controlled trial (three arms) T1: rTMS T2: NMES T3: traditional therapy	1 Hz rTMS (100 % rMT)	1200 pulses (20 min)	Unaffected	Pharyngeal	-	5 days/week, 2 weeks	Decrease in functional dysphagia severity and decrease of AP after rTMS
[67]	4 (2 male) 71 yoa	Hemispheric and sub-hemispheric stroke	Case series	5 Hz rTMS (90 % rMT)	3000 pulses/session	Site with minimum intensity to elicit MEP	‘Tongue’ hotspot	70 mm figure 8	5 days /week, 2 weeks	Improvement in videofluoroscopy measurements and quality of life after real rTMS.

The several parameters in these studies shown in this table indicate the differences between studies in the literature

*n* number, *yoa* years of age, *T* treatment, *RCT* randomised controlled trial, *rMT* resting motor threshold, *MEP* motor evoked potential, *VFS* videofluoroscopy, *AP* aspiration-penetration, *LMI* lateral medullary infarct, *BI* brainstem infarct, *TBI* traumatic brain injury, *PES* pharyngeal electrical stimulation, *PAS* paired associative stimulation, *LH* left hemisphere, *RH* right hemisphere, *NMES* neuromuscular electrical stimulation

**Fig. 1:**
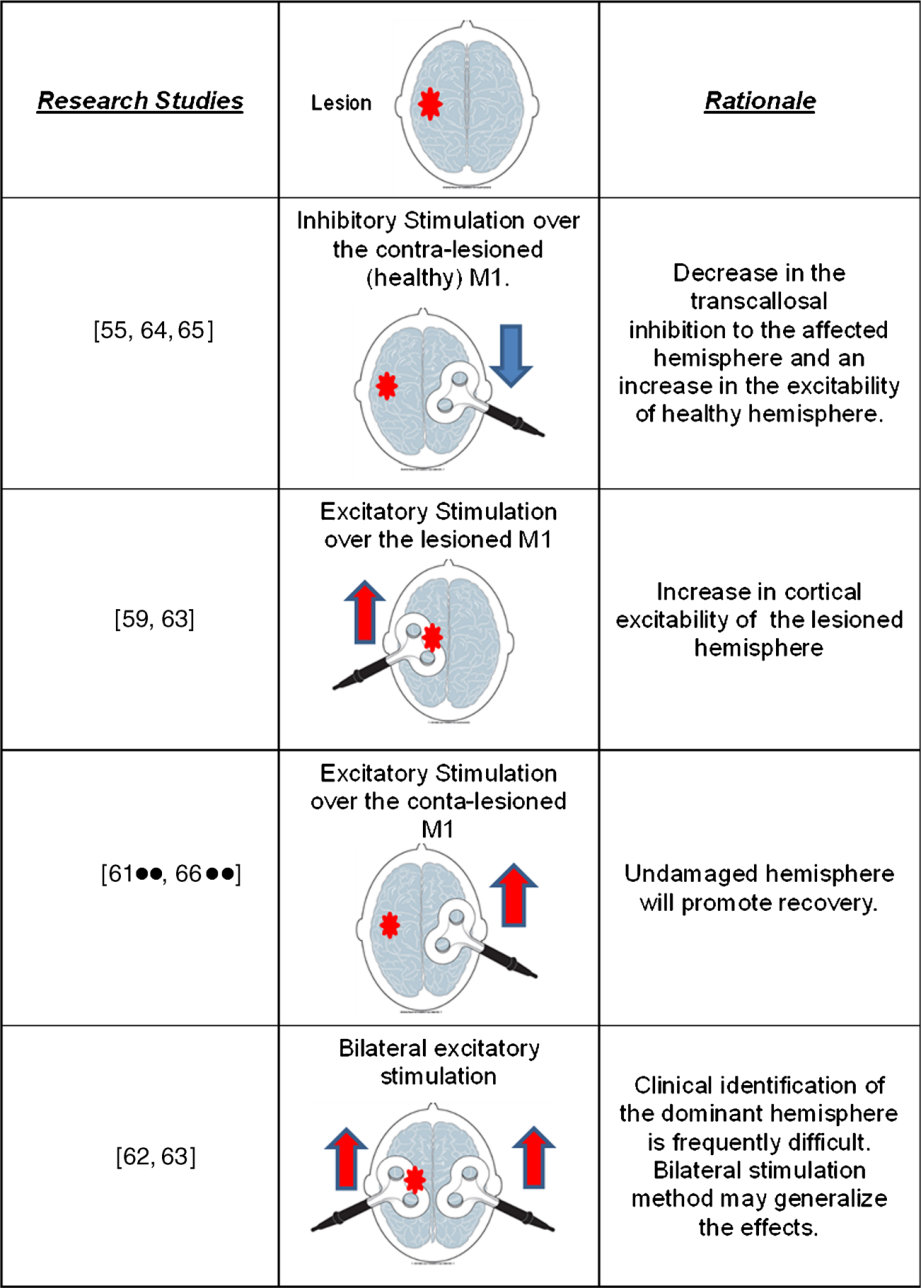
Studies using rTMS on dysphagic stroke patients. The rationale for using either excitatory (*red upwards arrow*) or inhibitory (*blue downwards arrow*) over the lesioned or unlesioned (lesion marked with a *star*) is shown in the *third column*

Even though only the effects of single sessions of rTMS have been investigated in health, several studies have used either excitatory [59, 60, 61••, 62, 63] or inhibitory [55, 64, 65] rTMS treatment regimen in stroke patients with dysphagia repeatedly over a different number of days with few exceptions [66••]. Also of interest is that fact that there are differences in the rationale behind the target selection (lesioned vs. unlesioned cortical representation) to apply the stimulation. Figure 1 shows the different protocols and the rationale used in the literature. In addition, different cortical musculature representations, i.e. representations of upper oesophageal sphincter [60], mylohyoid [55, 65], pharyngeal [61••, 66••], have been targeted with varying parameters or intensities.

As already reviewed in the meta-analysis [57], there are four randomised controlled trials (RCTs) in the literature investigating the effects of the rTMS on dysphagic stroke [60, 61••, 65, 66••]. The pooled effect size showed a moderate significant overall effect size, favouring the use of rTMS over the cortical representation of musculature involved in swallowing in stroke patients. However, the issue here is that these studies had several differences in target cortical representation (mylohyoid, pharyngeal, oesophageal), time post-stroke recovery phase of the patients studied, therapeutic regimens (treatment repeats) and hemispheric application with respect to the lesions.

Moreover, there seems to be variability in the outcome measures used in the research studies. These outcome measures for the effect range from videofluoroscopy and direct visualisation of changes in physiology [65, 66••] to self-rated dysphagia awareness measures [60].

Last but not least, patient characteristics differed across studies. Stroke type (ischemic and haemorrhagic, hemispheric and brainstem) and time post-onset (combining acute and chronic stroke patients) are just a few of the diverse variables that preclude direct comparisons.

## Conclusions

Repetitive TMS in health and swallowing disorders has provided valuable information towards further understanding of the swallowing network and its capacity to change for beneficial swallowing outcomes.

In health, rTMS was employed as a means to unravel the connectivity and the ‘flexibility’ of the swallowing network. Studies in health inform evidence-based decisions about the optimal frequency and intensity amongst other parameters of the stimulation protocols. Most importantly, results from studies with a translational component, such as inhibitory rTMS in health, assist in identifying the optimal target locations prior to the use of the neurostimulation technique as a treatment for dysphagia. Currently, the use of rTMS in dysphagia post-stroke seems to hold promise for beneficial changes in behaviour, but no large (or multicentre) randomised controlled study has yet been performed. To date, all the published studies with rTMS have targeted dysphagia post-stroke. Applying neurostimulation approaches to different disease aetiologies and accounting for several factors (age, lesion type, time from diagnosis), while measuring neurophysiological and functional outcome measures, will provide us further information about the endogenous plastic changes in humans with regard to swallowing function.

Nevertheless, there are profound differences in studies utilising rTMS in stroke dysphagic populations when considering the research methodologies utilised by different groups. Comparisons between the studies are difficult since most of the studies have applied rTMS on different targets (lesioned vs. contralesioned hemisphere) and different muscle groups (mylohyoid, pharyngeal, oesophageal).

Given the evidence that the mechanisms underlying the effects of rTMS range from changes in neuronal excitability to changes in neurotransmitter concentrations along with the effect of genetic predisposition of responders vs. non-responders to neurostimulation, we conclude that further work should be performed in the field.

It is important to continue research into neurostimulation techniques for swallowing rehabilitation for two reasons. Firstly, there is a potential avenue for clinical utility of neurostimulation in dysphagia rehabilitation clinics. Secondly, by studying how we can modulate the swallowing network, the optimal time window for swallowing modulation and the exact neurophysiological and behavioural effects of neurostimulation, we will be able to accumulate a greater knowledge about the adaptive changes that we can promote to our patients.

To conclude, recent research studies investigating the effects of rTMS for dysphagia rehabilitation have shown promising results. There is some paucity that this neurostimulation technique will be viewed as powerful tool in the hand of a rehabilitation clinician in the future. However, currently, the field of neurorehabilitation science in dysphagia is diverse in nature and methodological differences across research studies are accentuating the need for further investigations.
